# Gridded livestock density database and spatial trends for Kazakhstan

**DOI:** 10.1038/s41597-023-02736-5

**Published:** 2023-11-29

**Authors:** Venkatesh Kolluru, Ranjeet John, Sakshi Saraf, Jiquan Chen, Brett Hankerson, Sarah Robinson, Maira Kussainova, Khushboo Jain

**Affiliations:** 1 Department of Sustainability and Environment, University of South Dakota, Vermillion, SD 57069 USA; 2 Department of Biology, University of South Dakota, Vermillion, SD 57069 USA; 3 Department of Geography, Environment, and Spatial Sciences, Michigan State University, East Lansing, MI 48823 USA; 4 Center for Global Change and Earth Observations, Michigan State University, East Lansing, MI 48823 USA; 5 Leibniz Institute of Agricultural Development in Transition Economies (IAMO), Theodor-Lieser-Str. 2, 06120 Halle (Saale), Germany; 6 Institute for Agricultural Policy and Market Research & Centre for International Development and Environmental Research (ZEU), Justus Liebig University, Giessen, Germany; 7 Kazakh National Agrarian Research University, AgriTech Hub KazNARU, 8 Abay Avenue, Almaty, 050010 Kazakhstan; 8 Kazakh-German University (DKU), Nazarbaev avenue, 173, 050010 Almaty, Kazakhstan

**Keywords:** Population dynamics, Grassland ecology, Ecological modelling

## Abstract

Livestock rearing is a major source of livelihood for food and income in dryland Asia. Increasing livestock density (LSK_D_) affects ecosystem structure and function, amplifies the effects of climate change, and facilitates disease transmission. Significant knowledge and data gaps regarding their density, spatial distribution, and changes over time exist but have not been explored beyond the county level. This is especially true regarding the unavailability of high-resolution gridded livestock data. Hence, we developed a gridded LSK_D_ database of horses and small ruminants (i.e., sheep & goats) at high-resolution (1 km) for Kazakhstan (KZ) from 2000–2019 using vegetation proxies, climatic, socioeconomic, topographic, and proximity forcing variables through a random forest (RF) regression modeling. We found high-density livestock hotspots in the south-central and southeastern regions, whereas medium-density clusters in the northern and northwestern regions of KZ. Interestingly, population density, proximity to settlements, nighttime lights, and temperature contributed to the efficient downscaling of district-level censuses to gridded estimates. This database will benefit stakeholders, the research community, land managers, and policymakers at regional and national levels.

## Background & Summary

Livestock grazing is a significant and widespread land use practice in drylands. Approximately 2.4 billion heads of livestock are distributed worldwide, providing livelihood to one billion people that earn $2 per day or less^1^. Population explosion, dietary shifts from plant- to animal-based consumption, globalization, and other social-environmental changes have increased the demand for meat and dairy products in dryland Asia^2,3^. This dramatic increase in livestock numbers has increased the scale and intensity of grazing, reshaping the grassland ecosystem’s species composition, structure, function, and dynamics^4^. Furthermore, livestock grazing systems have a broader, bi-directional interaction with climate change (CC). CC is widely characterized by drought and heat stress, causing loss of livestock production, feed availability (quality and quantity), and uneven distribution of water resources and disease vectors^5^. Meanwhile, the intensification of livestock densities and disturbances such as overgrazing cause environmental changes such as greenhouse gas emissions, elevated albedo, alterations in nitrogen and phosphorus cycles, and loss of biodiversity and ecosystem services^6–8^. However, unlike climate change, grazing impacts can be managed and regulated^9^, especially with a detailed assessment of livestock impacts^10^.

Rangelands facilitate various ecosystem functions and services apart from food that are non-provisioning in nature. Globally, rangelands have a long land-use history with species-rich vegetation communities that can store ~15% of the global organic carbon, protect soil fertility, prevent soil erosion, and influence the hydrologic cycle^11^. However, rangelands are also experiencing significant land cover changes and shifts, abandonment, and fragmentation due to livestock overgrazing and climate change^12,13^. Kazakhstan (KZ) is an interesting case study as these rangelands have historically undergone significant land-use change due to grassland conversion to cropland and intensifying livestock grazing before the reversal of these processes since the 1990s^14,15^. Previous studies have shown that 76.1% (2000–2015) of KZ is sensitive to desertification due to overgrazing, grassland conversion, mining, and infrastructure development and spends ~$5.4 billion annually to combat degradation^16,17^. However, a lack of longitudinal gridded livestock density (LSK_D_) datasets at the grid/county level has limited national-scale investigations of livestock impacts on terrestrial ecosystems. Livestock census data for KZ at the district level can be accessed from the Ministry of Agriculture or the national statistical agency websites. However, obtaining sub-district or village-level livestock numbers in KZ remains challenging. In addition, information on livestock farm locations, size and type, herd composition, production potential, and consumption behaviors (milk, meat, or wool) are not easily accessible. These shortcomings necessitate the development of high spatiotemporal gridded LSK_D_ maps that enable precise livestock management, decrease environmental impacts, and use for a range of research and policy implications, from epidemiology and economics to environmental science^7,8^. The LSK_D_ distribution further helps explain livestock systems and their environmental performance, determine spatial hotspots, and develop plausible strategies for environmental improvement^18,19^.

Livestock census data are generally publicly available only at the provincial/national level for most countries and lack timeliness^20^. These census data can be considered approximate ‘truth’ within the district/province; however, they fail to provide detailed information on the geographical distribution of livestock densities^21^. Hence, previous studies have aimed at developing gridded LSK_D_ datasets at global, continental, national and regional scales, with various resolutions and animal species^8,19,21–26^. The Gridded Livestock of the World dataset (GLW), initially developed in 2007, with the most recent iteration (GLW-4) in 2022, produced global maps of cattle, buffaloes, horses, sheep, goats, pigs, chickens, and ducks for 2015 at a spatial resolution of 5 arcminutes (~10 km at the equator)^27^. GLW-4 uses dasymetric (DA) and area-weighted (AW) approaches to disaggregate statistical counts to gridded datasets^8,28^. Previous studies have mentioned that the accuracies of GLW are lowest in semi-arid and low-population regions due to the underrepresentation of livestock numbers that move seasonally across remote and inaccessible areas^7,8,15^. In addition, the GLW dataset was developed using livestock census data from first-level (provincial) administrative divisions for most countries (Russia, Kazakhstan, Mali, Sudan, and Saudi Arabia). The coarse resolution, lack of temporal spans and validation severely limit the suitability of the GLW for regional and local studies^8,21^. To complement the GLW project, Meisner *et al*.^5^ developed a longitudinal (2000–2020) gridded cattle and pig density dataset for Malawi and Uganda with a resolution of 0.017° (~1.88 km), and Li *et al*.^21^ developed cattle and sheep density datasets at five-year intervals. However, to our knowledge, no single study has developed high-resolution (1 km), multi-temporal small ruminant (sheep and goat) and horse density estimates spanning two decades for any region worldwide.

To fill these knowledge gaps, we aimed to develop gridded LSK_D_ estimates for every year from 2000 to 2019 at 1 km resolution for KZ, facilitating their use in longitudinal analyses. Developing a small ruminant and horse density database is particularly important for Kazakhstan, as they significantly contribute to meat production, the pastoral economy, and land cover shifts. Mapping small ruminant densities provides insights into their extent and areas of concentration. Furthermore, mapping horse densities that have significant energy and nutritive elements assists in energy and fodder resource planning, thereby supporting their well-being and productivity, especially in regions of cultural and economic importance^29^. These databases can further assist policymakers and land managers in developing targeted land use, pasture management, and carrying capacity strategies. Developing high-resolution gridded estimates from statistical census data requires the integration of various forcing mechanisms of a social-environmental system (SES) (hydroclimatic and socioeconomic) to meet the requirements of spatial calculations, methods, and models. We seek to address the following research questions by developing the LSK_D_ estimates: 1) What are the spatiotemporal distributions and trends of LSK_D_ (small ruminants and horses) across KZ during the study period? 2) Do LSK_D_ hotspots capture/follow settlement patterns? and 3) Which SES drivers are significant in explaining the LSK_D_ distribution across KZ? To address these research questions, our objectives were to (i) employ a random forest (RF) regression model to disaggregate district-level livestock numbers into gridded estimates of LSK_D_, (ii) develop a high-resolution (1 km) gridded LSK_D_ spatial database for small ruminants and horses in the KZ during 2000–2019 using vegetation proxies, climatic, socioeconomic, topographic, and proximity variables, (iii) detect spatiotemporal trends using Mann-Kendall and Sen’s slope approaches, and (iv) adjust the predictions with observed census data and validate them with maps from previous literature.

## Methods

### Study area

**Fig. 1:**
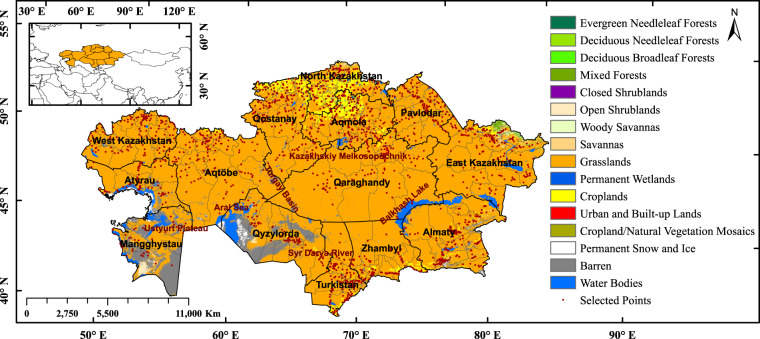
Land cover map of Kazakhstan overlaid with selected sample points, provincial boundaries (black), and district boundaries (grey). The map was derived from MODIS MCD12Q1 land cover product for 2020.

Kazakhstan, located at the center of Eurasia, is the largest landlocked country with an area of 2.72 million km^2^. It has 17 regions (oblasts) and 215 districts (rayons), with an estimated population of 19.2 million and a gross domestic product of 220.5 billion dollars (Fig. 1). Grasslands cover 85.4% of KZ (Fig. 1), followed by barren (5.79%), croplands (4.2%), water (2.73%), savannas (0.66%), and forests (0.33%)^30^. Most of these grasslands are in semi-arid regions receiving average annual precipitation of 100–300 mm/year (often as snow than rain)^31^. The KZ rangelands span across large elevation gradients (from −227 to 3000 m) and cover multiple ecological biomes, from the desert ecosystems (dominated by woody shrubs and ephemeral spring bulbs) to short and tall-grass steppes on the plains and alpine meadows^32^. The climate is extremely continental with fluctuating rainfall patterns, hot, dry summers (average temperature of 29 °C during June-August), and very cold snowy winters (average temperature of −20 °C during December-February)^33^. *Stipa*, *Festuca* and *Artemisia* dominate the grassland vegetation with fertile Chernozems soils in the northern part of KZ^34^. Before the Soviet Union collapsed in 1991, agricultural lands were generally used for growing cereals in the north and for livestock grazing in the south. After 1991, the country lost two-thirds of its livestock and one-third of its cropland. Since 2000, KZ has experienced a 15% recovery of abandoned croplands and a 30% increase in livestock numbers (compared to 1998)^14^. The livestock in KZ are raised in three different farm types: (i) agricultural enterprises with a few thousand to tens of thousands of animals, (ii) private farms with tens to thousands of livestock and (iii) household farms with only a few livestock. While 60% of grazing livestock are raised on household farms, private farms are the fastest-growing farm types and differ the most in size and grazing practices^15^. Notably, private farms and enterprises can legally lease pastures on state lands, whilst households have legal access only to kitchen gardens and village pastures available to all residents.

### Social-environmental system (SES) database

The spatial heterogeneity of resources and environmental factors have a significant impact on the distribution of livestock. Hence, based on the existing literature, we identified and obtained 13 SES factors that may potentially influence LSK_D_ distribution^21,24,35–37^. The variables with global extents were selected to facilitate the replication of the current study in other geographic regions (Table 1). Net primary productivity (NPP) and leaf area index (LAI) were derived from moderate-resolution imaging spectroradiometer (MODIS) sensors^38,39^. Annual precipitation, air temperature and vapor pressure deficit data were computed from climatologies at high resolution for the earth’s land surface areas (CHELSA) dataset^40^. Soil moisture, snow depth and net downward shortwave radiation were obtained from the famine early warning systems network (FEWS NET) land data assimilation system (FLDAS) dataset^41^. Elevation data was obtained from the shuttle radar topographic mission (SRTM)^42^. The location and temporal distribution of surface water were obtained from the joint research center (JRC) yearly water classification history, v1.4 dataset^43^. Pixels with permanent water bodies were extracted from this dataset, and Euclidean distances were calculated to derive the distance to water body maps for each year. Settlement locations were downloaded from the Geofabrik website, and Euclidean distance was calculated to derive the distance to the settlement map. The settlement shapefile was extensively corrected using imagery from OSM, Bing, Google, and Yandex, national census data, administrative boundaries maps, and Wikipedia articles containing coordinates to ascertain the locations of settlements. Annual population density estimates were downloaded from the Worldpop website^44^. Nighttime light data was obtained from the defense meteorological satellite program (DMSP) nighttime lights extension dataset^45,46^. Most of these datasets were downloaded through Google Earth Engine for 2000–2019 but rescaled to annual and 1 km spatial resolution.

**Table 1:** Description and data sources of the predictor variables considered in the study.

Variable	Source	Units (resolution)	Temporal resolution
Annual precipitation	Climatologies at high resolution for the earth’s land surface areas (CHELSA)	mm/year (1 km)	1979–2019 (monthly)
Annual average temperature		°C (1 km)	
Vapor pressure deficit		KPa (1 km)	
Solar radiation	FLDAS Noah Land Surface Model L4 Central Asia Daily 0.01 × 0.01 degree	W/m^2^ (1 km)	2000 – present (daily)
Soil moisture		m^3^/m^3^ (1 km)	
Snow depth		cm (1 km)	
Population density	WorldPop	No/km^2^ (1 km)	2000–2020 (annual)
Nighttime light	Version 4 DMSP-OLS Nighttime Lights Time Series and VIIRS Nighttime Day/Night Band Composites Version 1	Averaged DN (1 km)	1992–2021 (annual)
Elevation	SRTM DEM (SRTMGL1_v003)	m (30 m)	2000
Distance to settlements	Geofabrik website http://download.geofabrik.de/	m (1 km)	NA
Distance to waterbodies	JRC Yearly Water Classification History, v1.4	m (30 m)	1984–2021 (annual)
Leaf area index	MOD15A2H.061 & MCD15A3H.061 MODIS Leaf Area Index/FPAR	M^2^/m^2^ (500 m)	2000–2023 (8-day)
Net primary productivity	MOD17A3HGF.006 & MOD17A3HGF.061 Terra Net Primary Productivity	Kg *C/m^2^ (500 m)	2000–2023 (annual)
Small ruminants and horses	Kazakhstan Bureau of National Statistics https://stat.gov.kz/	Livestock heads (NA)	2000–2019 (annual)

NPP and LAI were chosen because they indicate forage biomass availability, which varies geographically and supports livestock^47^. Environmental variables, such as precipitation, temperature, solar radiation, and vapor pressure deficit, were included to reflect climatological differences across regions. These environmental variables directly affect vegetation growth and indirectly impact livestock distribution by determining feed and water availability in a given area^36,37,48^. Additionally, the distance to settlements and water bodies was included as high-productive forage close to residential areas, and water bodies are more convenient for herders to allow livestock to graze^21,37^. Elevation was selected as it affects biotic variables, such as plant species composition and production, affecting livestock grazing distribution and movement^49,50^. Population density and nighttime lights were also considered as livestock are generally clustered around settlements with human populations^15^. Although the variables selected were not comprehensive, they were highly representative and reflected various heterogeneous aspects of livestock distribution.

District-level livestock numbers for small ruminants (i.e., sheep & goats combined) and horses were obtained from the Bureau of National Statistics, KZ (Table 1) for 2000–2019. Two years (2000–2001) of livestock statistics are missing for districts in Aktobe Oblast, and one year (2000) is missing from districts in Atyrau Oblast. The statistics for these missing years were linearly interpolated from the year before (1999) to the year after (2002 and 2001, respectively). District shapefiles were obtained from the database of the global administrative areas (GADM) website (Table 1). LSK_D_ estimates were computed by dividing the livestock numbers with the district-level areal estimates. These LSK_D_ estimates computed across years were assigned to the district shapefiles and were converted to rasters with a 1 km resolution for 2000–2019. Due to the geopolitical restructuring of KZ’s administrative districts during the study period, numbers from several affected districts were summed to generate a final database of 200 LSK_D_ sample points per year.

### Gridded livestock density: predictions

During Soviet times, the human population was settled in permanent villages with structured and strictly regulated livestock migration (grazed in large, transhumant, communal herds) across the pastures^15^. Fodder was provided under subsidized networks, especially during winter, to supplement livestock. After the Soviet Union collapsed, long-distance migration was immobilized, with the human population remaining sedentary and livestock housed primarily on small household farms^51^. This immobilization caused overgrazing around settlements, while outposts farther away fell into disrepair^14,34,52^. The livestock housing unit locations or sub-district level livestock data are required to capture these livestock density patterns around settlements. Lack of these datasets and, based on personal observation and expert guidance, we assumed that settlement locations could serve as proxies to capture grazing distribution near settlements.

Our primary objective is to enhance the sampling strength from 200 to 2000 points to train and test the machine learning model effectively. This can be achieved by (i) selecting ten random points or (ii) ten settlement locations across each district. In either case, we assign the same livestock density value to the 10 points per district, as we do not have data at a higher resolution than the district level. We chose option two of selecting ten settlement points with a higher human population from the available 100 or 1000 settlement points in each district. Therefore, we first obtained settlement locations (i.e., city, hamlet, town, and village) across KZ and tagged the human population counts for each settlement location to capture the LSK_D_ distribution around settlements. We then chose the top 10 settlement locations per district by the human population, increasing the sampling strength to 2000 samples for each year. The selected 2000 settlement points cover hamlets, villages, towns, and cities with a human population ranging from 10 heads to 1.36 million heads. Of these 2000 settlement locations, 87% of the points are from rural areas (336 hamlets and 1412 villages), and the rest from towns and cities. These 2000 settlement location points spread across KZ were used to extract the training and testing data from the livestock and environmental variables. While the settlement location is the same across years, the predictor and response variable values vary across years, allowing us to capture the changing livestock density values. The 2000 sample points are extracted based on three main assumptions, namely, 1) settlement locations are proxies of livestock housing units, 2) higher population settlements would have higher livestock density than other settlements in the district, and 3) ten settlement locations would suffice RF model training and testing.

**Fig. 2:**
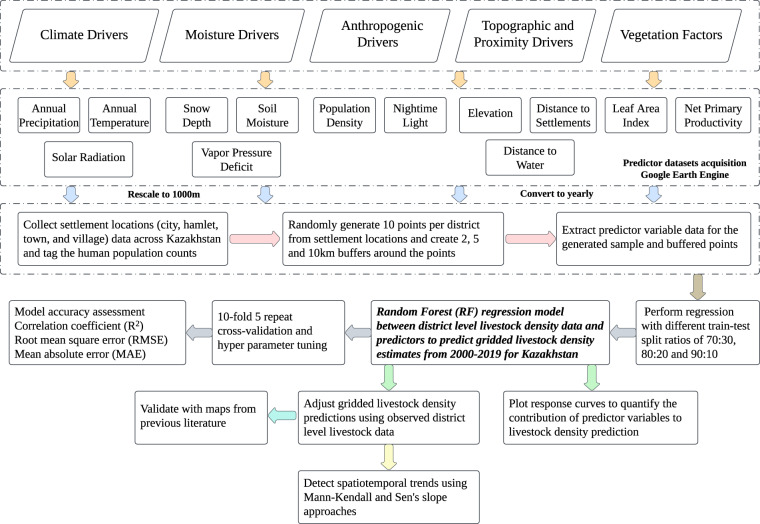
Schematic diagram of workflow for predicting livestock density with potential socioeconomic-environmental variables using district-level livestock census data with random forest regression model for 2000–2019 in Kazakhstan.

We first developed 12 RF models for predicting small ruminant density in 2015 with (i) 2000 samples each from the settlement locations and their respective buffers (2, 5 and 10 km radius), and (ii) train-test split ratios of 70:30, 80:20 and 90:10 for accurate estimates of gridded LSK_D_. The buffer distances were selected based on literature to account for daily livestock movements while disaggregating the district-level to grid-based estimates^15,52–56^. The sample points from the settlement locations and its three buffered polygons (2, 5 and 10 km) were used to extract data from the livestock rasters and 13 predictor variables to train and test an RF model (Fig. 2). Once the RF model was trained and tested with the livestock and predictor variables of the sampling data, it was applied on the predictor variable rasters obtained for 2015 to develop spatial livestock density map for 2015. The RF model-derived small ruminants map for 2015 was validated with the GLW dataset and maps from previous literature^8,47,57,58^.

Previous studies have also shown that RF proved efficient at predicting spatial patterns of species distribution compared to traditional linear models^19,59–62^. RF integrates multiple decision trees and activates attribute selection during training, alleviating over-fitting that generally occurs in conventional machine learning algorithms^63^. The closest parallel approach to our study is the development of GLW and Worldpop datasets that implemented a similar downscaling approach and proved that RF provides better predictions than land-use-based downscaling approaches^8,19,44,64,65^. These studies are very similar in their objectives to downscaling human/livestock population census data to the grid/pixel level. In addition, the RF model has also been widely implemented to downscale spatial resolution of various climate and vegetation datasets^66–68^.

Mann-Kendall (MK) and Sen’s slope (SS) trends were computed to examine the positive (increasing) or negative (decreasing) LSK_D_ trends from the model predictions^69,70^. More details regarding MK and SS trends can be found in Venkatesh *et al*.^31^. The buffer distance and data split ratio were obtained from the best-performing RF model (out of 12 models) that accurately captured the patterns and density values. While the data split ratio and buffer distance were held constant for all years, hyperparameter tuning, training, and testing of the RF model were performed independently for each year (Fig. 2).

Hyperparameter tuning was performed by optimizing the number of randomly drawn candidate variables (mtry) and the number of trees (ntree) to minimize errors and improve the model accuracy. We implemented 10-fold cross-validation with five repetitions to optimize the hyperparameters and select the best subset model^71^. The coefficient of determination (R^2^), root mean square error (RMSE), and mean absolute error (MAE), were evaluated during the training and testing phases to test the model performance (Fig. 2). We computed variable importance to identify the top predictors contributing to LSK_D_ prediction. We further developed response curves to understand the influence of biophysical and socioeconomic factors on LSK_D_ distributions. The response curves were created by plotting mean predicted livestock densities against the corresponding values of a particular covariate, where other covariates in the model were held constant at their median value^62^.

### Adjusting livestock density predictions

Predictions from the ML models need to be adjusted because downscaling might not accurately simulate identical livestock density values at the district and grid scales. For each district, the pixel values were multiplied by the ratio of observed livestock density estimates from census data to the sum of pixel values in the district obtained from the RF model. The livestock density adjustment was performed each year using the following equation^8,21^.

1 $$Ad{j}_{i}={P}_{i}\times \frac{{O}_{j}}{{P}_{j}}$$

Where i represents a pixel or grid and j represents an administrative district. Adj_i_ is the adjusted livestock density value of grid i, and P_i_ is the predicted livestock density value of grid i. O_j_ is the observed total livestock density in administrative district j, and P_j_ represents the predicted gridded livestock density of the corresponding administrative district.

## Data Records

The developed database is publicly available for download from the *figshare* repository (10.6084/m9.figshare.23528232)^72^. The repository has three folders with small ruminants, horses, and sample code and data in separate folders. Each file is saved in GeoTiff format with 1-km spatial resolution and an Albers equal-area conic projection. Each file is saved with an acronym of ‘sr’ for small ruminants (Sheep & goat) and ‘hr’ for horses, followed by an underscore and a year. Missing data are represented by “No data.”

## Technical Validation

### Spatiotemporal changes in livestock density

We implemented the RF models to estimate LSK_D_ at 1 km^2^ resolution for KZ. Each RF model was initially trained and tested with different data split ratios and buffered distances for small ruminant density data in 2015. We computed the independent test score, which is more indicative of the model generalization ability because it was not used in training, and the cross-validation (CV) score, which is more representative because it reflects the model performance on 80% of the data rather than only the 20% used for testing (Table 2). The RMSE/MAE of the CV score dropped when the RF model was developed using a 90:10 data split ratio instead of 70:30 for settlement, 5 km, and 10 km buffered locations but increased for 2 km buffer distances (Table 2). Similarly, the RMSE/MAE test scores decreased with a 90:10 data split ratio for settlement and 2 km buffered locations but increased for 5 km and 10 km buffering distances (Table 2). When RF was tested with a 90:10 split ratio, R^2^ increased for the 5 km and 10 km buffered datasets for test scores and all buffering distances for CV scores (Table 2). Overall, the RF model performed best with 10 km buffered data and a 90:10 data split ratio, with low RMSE/MAE and a high R^2^ (Table 2). Hence, this data split ratio and buffer distance were held constant for all years, and hyperparameter tuning, CV, training, and testing of the RF model were performed independently for each year to develop maps for other years (2000–2019) and livestock (horses and small ruminants).

**Table 2:** Cross-validation and independent test scores of random forest regression model for predicting small ruminants (sheep & goat) density with different buffer distances and data split ratios for 2015 (lower RMSE/MAE and higher R^2^ indicate better model fit).

Buffer distance	Cross-Validation score			Independent test score		
	RMSE	R^2^	MAE	RMSE	R^2^	MAE
**same location**						
70-30	70	0.49	17.29	84.17	0.48	20.93
80-20	65.3	0.59	16.74	87.21	0.29	19.29
90-10	68.96	0.63	17.08	50.29	0.21	14
**2 km buffer**						
70-30	52.22	0.51	13.4	62.96	0.7	12.35
80-20	61.09	0.51	14.46	22.98	0.59	8.65
90-10	57.13	0.54	13.13	20.33	0.68	8.38
**5 km buffer**						
70-30	49.55	0.6	11.26	30.2	0.33	7.09
80-20	45.63	0.62	9.9	35.77	0.25	7.9
90-10	44.07	0.6	9.3	35.67	0.38	7.22
**10 km buffer**						
70-30	21.11	0.62	5.84	11.13	0.79	4.74
80-20	19.45	0.67	5.4	12.68	0.73	4.94
90-10	17.53	0.76	4.87	11.75	0.82	4.74

The Cross-Validation and Independent test scores were computed between the observed (census data) and predicted (RF model) livestock density values using the training and testing datasets, respectively.

**Fig. 3:**
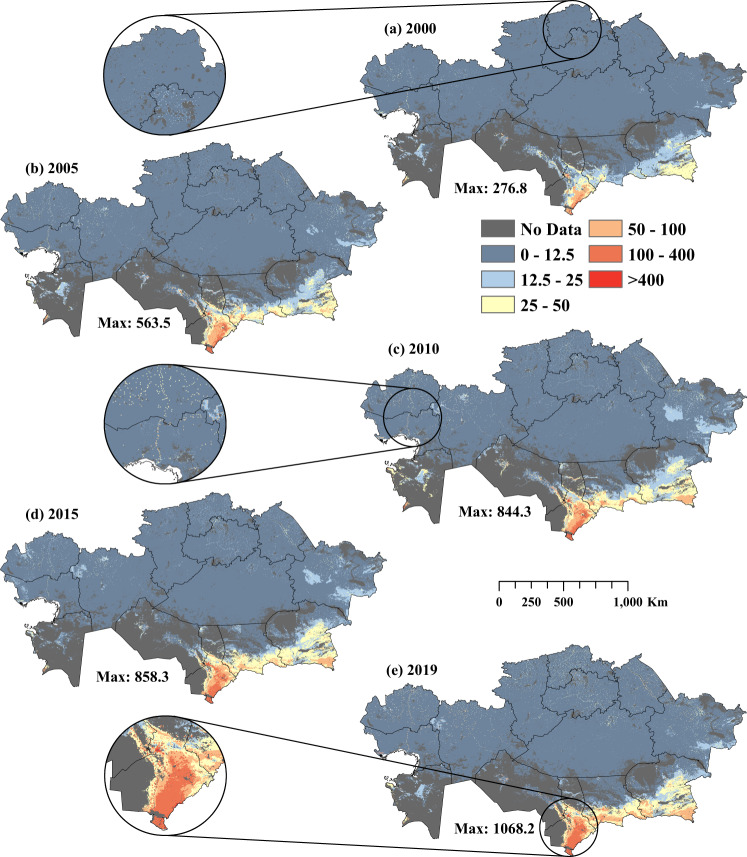
Spatial distribution of estimated small ruminant (sheep & goat) density across Kazakhstan for 2000–2019 (**a**–**e**). The enlarged circles show livestock density in three demonstration areas.

**Fig. 4:**
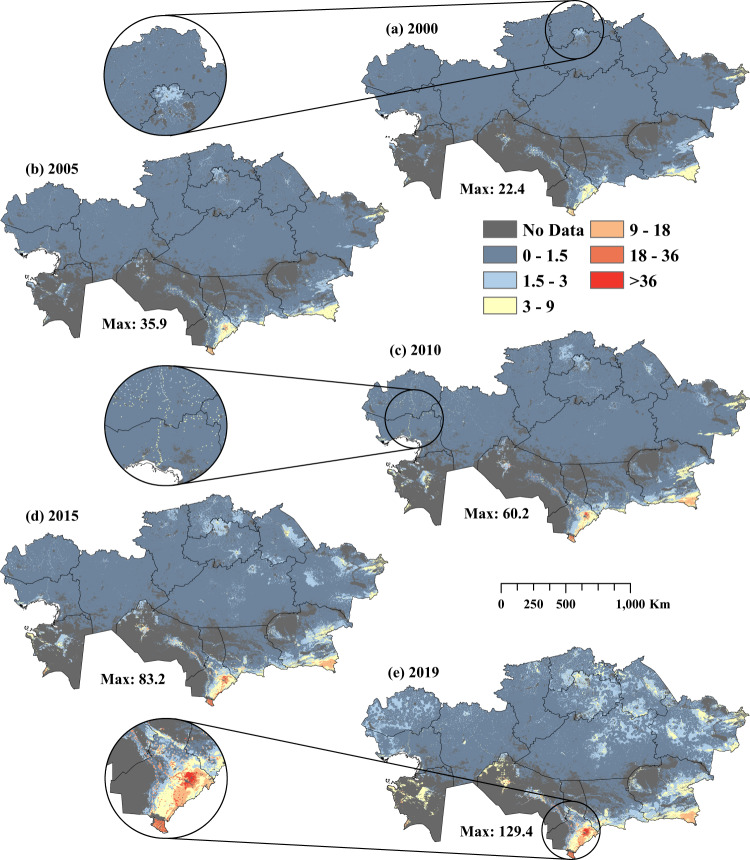
Spatial distribution of estimated horse density across Kazakhstan for 2000–2019 (a-e). The enlarged circles show livestock density in three demonstration areas.

**Fig. 5:**
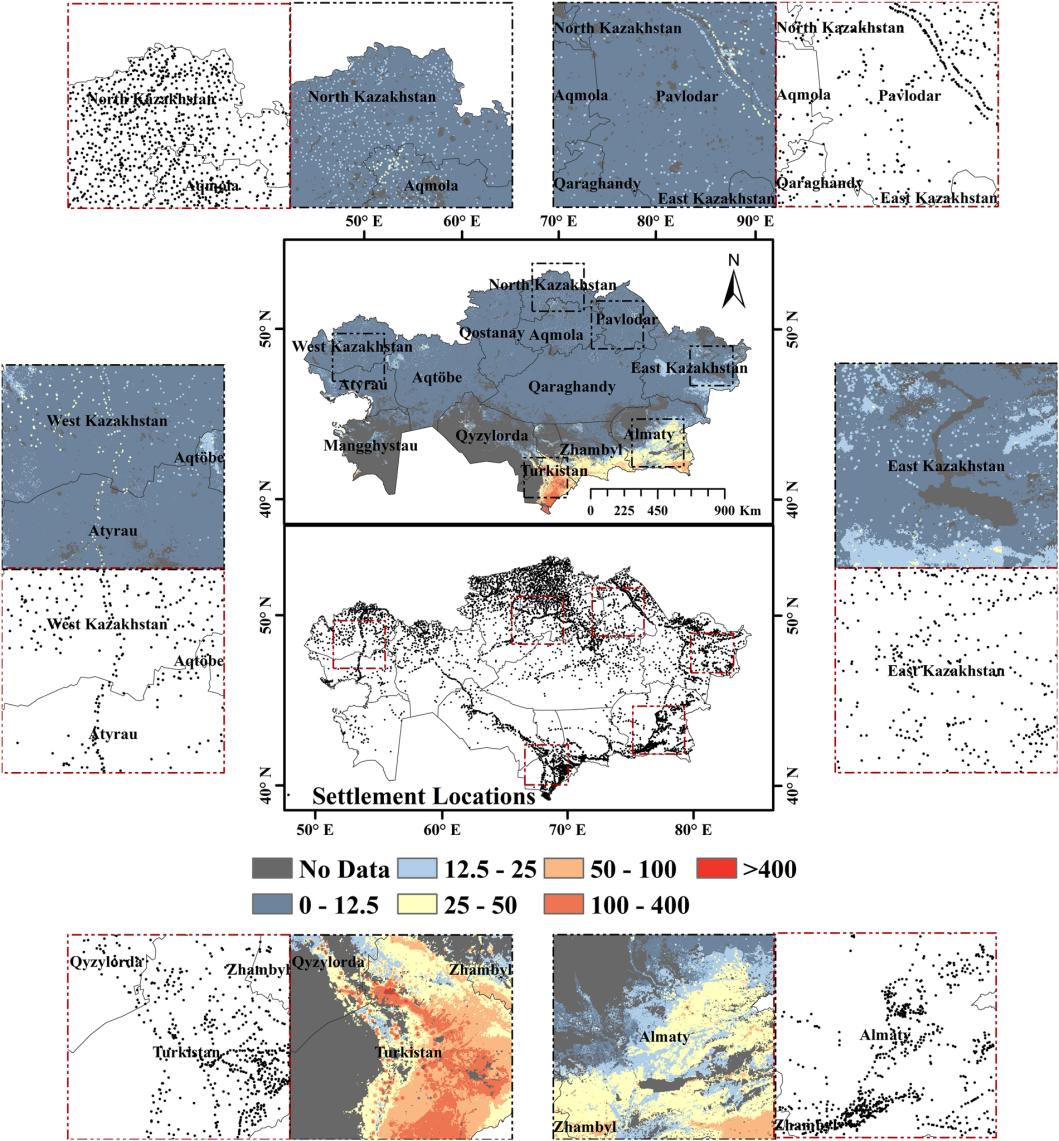
Spatial distribution of estimated small ruminant (sheep & goat) density based on random forest regression models that have a 90:10 split ratio and 10 km buffer distance for Kazakhstan in 2015. The enlarged portions with black borders show predicted livestock density distributions in six demonstration areas of Kazakhstan. The enlarged portions with red borders show settlement locations (n = 7234) obtained from the Geofabrik website in six demonstration areas of Kazakhstan. The settlement locations were extensively corrected using imagery from OSM, Bing, Google, and Yandex, national census data, administrative boundaries maps, and Wikipedia articles containing coordinates to ascertain the locations of settlements.

Gridded LSK_D_ databases for 2000–2019 were developed (Supplementary Figs S1, S6), fit statistics (R^2^, RMSE and MAE) were calculated (Supplementary Table S1), and maps with 5-year intervals were produced (Fig. 3, 4). The predicted database was adjusted using the livestock density adjustment method to ensure that the cumulative values of the pixels within a district match the total livestock density recorded in the census database for a specific year. The fit statistics for small ruminant density estimates showed that RMSE ranged from 5.16 to 18.12, MAE from 2.34 to 4.76, and R^2^ from 0.63 to 0.94 for 2000 to 2019 (Supplementary Table S1). Similarly, for horse density estimates, RMSE ranged from 0.43 to 2.86, MAE from 0.21 to 0.78 and R^2^ from 0.54 to 0.8 for 2000 to 2019 (Supplementary Table S1). Higher LSK_D_ hotspots were found in the southern and southeastern regions of KZ, namely, Turkistan, Zhambyl and Almaty, for all years (Supplementary Figs S1, S6). Additionally, medium-density livestock hotspots were evident in the western and northern Kazakhstan regions, namely, West Kazakhstan, North Kazakhstan and Aqmola (Fig. 4, 5). The higher livestock densities evident as dots from enlarged patches indicate that the RF model allocated higher LSK_D_ values following settlement patterns (Figs. 3–5).

**Fig. 6:**
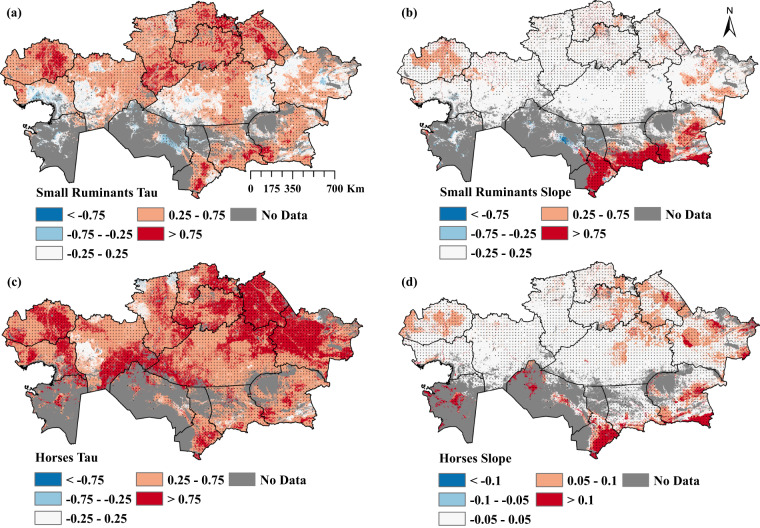
Spatial distribution of Kendall’s Tau and Sen’s slope for small ruminants (**a,b**) and horses (**c,d**) during 2000–2019. Dots indicate pixels with significant p-values (p < 0.05).

The MK test results for small ruminants and horses revealed significant (p < 0.05) positive trends across KZ with a smaller patch of negative trends in Qaraghandy, Qyzylorda, Almaty and Aqtobe regions (Fig. 6). Small ruminant, and horse density slope estimates ranged from −0.75 to 0.75 head/km^2^ and −0.1 to 0.1 head/km^2^ across the country, with the highest slopes in the southern (Turkistan, and Zhambyl) and southeastern (Almaty and East Kazakhstan) regions of KZ (Fig. 6). High, positive slopes were also found in Aqmola, Pavlodar, North Kazakhstan, West Kazakhstan, and Qaraghandy regions (Fig. 6).

### Validations

Mann-Kendall trends and Sen’s slope analysis showed that the reconstructed LSK_D_ estimates exhibited increasing trends and relative differences in rates of change in space across KZ for 2000–2019. These trends align well with previous findings that showed that KZ experienced an upward trend of sustained and steady growth in livestock densities from 2003^31,73–75^. Furthermore, the reconstructed LSK_D_ estimates were compared against the GLW (2010 and 2015), and off-take rate datasets (2015) developed for KZ (Supplementary Fig. S7). The off-take rate signifies the percentage of vegetation consumed by livestock, and higher off-take rate values indicate greater grazing activities. Visual validation with the GLW and off-take rate maps confirms that the reconstructed maps captured higher LSK_D_ in southern and southeastern Kazakhstan regions along with medium density values in northern and western regions (Supplementary Fig. S7). However, the predicted maximum GLW sheep & goat (167.5 and 164.6 for 2010 & 2015, respectively) and horse (7.4 and 6.6 for 2010 & 2015, respectively) density values are significantly lower than the observed census and our prediction results (small ruminants − 844.3 and 858.3 and horses − 60.2 and 83.2 for 2010 & 2015, respectively) (Supplementary Fig. S7). The underestimation in the GLW might be due to the lack of district-level data for the dataset development. Indeed, the GLW dataset developers have discouraged using the GLW in countries such as Russia, Kazakhstan, Mali, Sudan, and Saudi Arabia^8,21^.

Apart from GLW, Piipponen *et al*. (2022) developed global livestock carrying capacity maps for 2001–2015 and a relative stocking density (RSD) map for 2010 using GLW, NPP, slope, temperature, forest, and grassland cover as inputs^47^. Regional assessment through visual validation shows that the RSD for south-central and southeastern parts of KZ were categorized as overstocked, which matches our areas of high LSK_D_ estimates. In addition, northern and northeastern regions were categorized under medium pressure, while central and southwestern regions were categorized under low pressure, matching our LSK_D_ distributions. Though the units or maps were not directly comparable, the RSD was derived by dividing GLW livestock densities by the potential density that could be available based on grass biomass availability^47^. In another study, Erb *et al*. (2007) developed a grazing suitability map that categorized most regions of KZ in the lowest suitability class for grazing^57^. However, the few high-suitability regions (e.g., south and southeast) match our areas of high LSK_D_ clusters. A nationwide livestock survey was carried out by Schettino *et al*. (2021) to collect geographical coordinates of small ruminant holdings and livestock population counts in KZ during 2018–2019^58^. They georeferenced 2478 small ruminant holding locations, and visual validation confirms that the current LSK_D_ predictions align with those areas of livestock farm locations.

Animal husbandry accounts for 75% of all agricultural lands in KZ, which includes croplands and grasslands allocated to feed production^15^. The cultivated lands utilize agropastoral production systems, where agricultural waste can feed herbivorous livestock, promoting livestock breeding^2,76^. In addition, the grasslands utilize pastoral production systems that provide high-quality forage and space for raising livestock^21^. Our results align with these patterns as we found medium to higher livestock density concentrations in cultivated and grassland areas across all years in KZ. Medium-density livestock hotspots are found on cultivated lands in the northern and southern parts of KZ, as these regions have high grazing areas under agropastoral production systems. We also found high-density hotspots in pastoral production systems in the western plains and southern mountainous regions with high precipitation, snow, radiation, and temperatures that promote forage growth. In addition, the low-density livestock hotspots were spread across the country near settlements, as a vast area of KZ supports pastoral production systems.

We used a 10 km buffer radius for sampling the predictor datasets, which might induce spatial autocorrelation or clustering if suitable predictor variable values are available in neighboring pixels. The 10 km buffer captures livestock sedentarization around settlements and accounts for livestock that travel larger distances ( > 10 km). Moreover, lower buffer distances (2 and 5 km) did not result in better predictions, mandating using 10 km buffers. Additionally, spatial autocorrelation or clustering might occur as a stratified selective sampling strategy was used instead of stratified random sampling to sample predictor variables. Previous studies on livestock assessments have also detected spatial autocorrelation, clustering, or hotspots, which have helped to understand the characteristics of distribution and transition and to derive meaningful information for managing grasslands^23,77–79^. Clustering, despite potential contention, demonstrably represents genuine spatial dependence patterns inherent in the distribution of livestock. The identified hotspots can help herders and land managers devise proactive measures, village awareness programs, and on-the-ground actions for preventing livestock grazing in highly dense clusters and degraded areas^80^. The optimization of productive areas for grazing and identifying livestock density hotspots based on resource availability is a topic of ongoing scholarly discourse. Moreover, it serves as a basis for developing and implementing government policies in various regions^78^.

### Significant SES variables

**Fig. 7:**
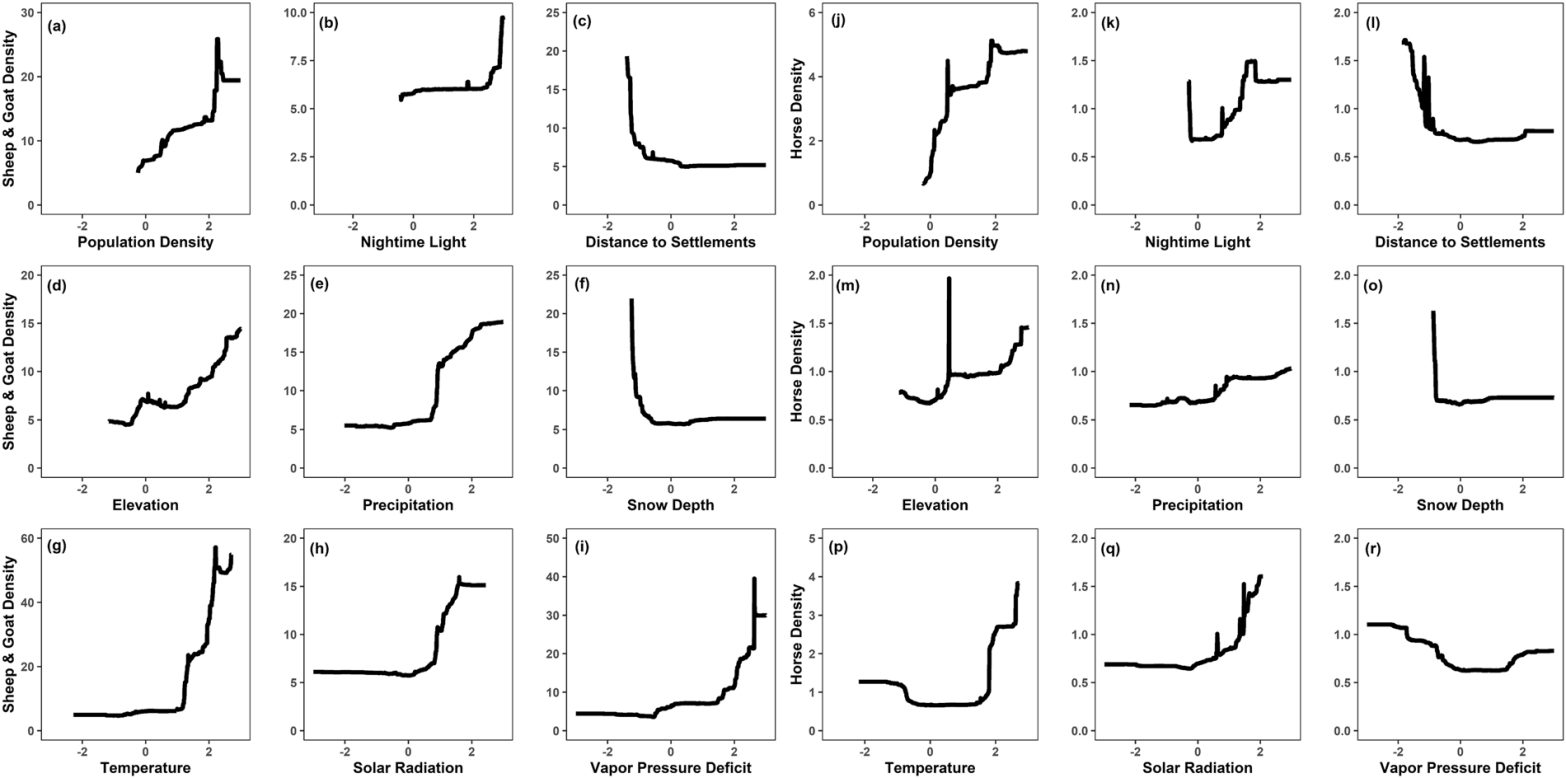
Response curves for the top-ranked covariates in predicting small ruminants (sheep & goat) (**a**–**i**) and horse density (**j**–**r**) with random forest regression models. The horizontal axis shows the standardized predictors, whereas the vertical axis shows livestock density.

Variable importance from the RF model showed that temperature, population density, distance to settlements, vapor pressure deficit, solar radiation, and nighttime light were the top contributing drivers for horse and small ruminant density distributions (Supplementary Fig. S8). Spatial distributions of the predictors were created to examine the patterns of allocated livestock spatial densities (Supplementary Fig S9). The RF model allocated higher LSK_D_ values where population density is higher and distance to settlements is lower. It also allocated higher livestock densities in the southeastern mountainous regions where precipitation and snow depth are high, temperature and vapor pressure deficit are low. (Supplementary Fig S9). Response curves were plotted for better visualization of the contribution of SES variables on LSK_D_ prediction (Fig. 7). We found that increasing population density, elevation, nighttime lights and decreasing distance to settlements showed increasing LSK_D_ (Fig. 7). We also found that increasing precipitation, temperature, solar radiation and decreasing snow depth increased LSK_D_ (Fig. 7). Furthermore, decreasing vapor pressure deficit increased horse density estimates and resulted in stable estimates for small ruminant density predictions. In contrast, increasing vapor pressure deficit increased small ruminant density estimates and contributed to stable estimates for horse density predictions (Fig. 7).

Our results showed that socioeconomic and climate-related factors efficiently explained the spatial LSK_D_ distributions in KZ. Previous studies have shown that precipitation and temperature significantly affect the amount and quality of forage, consequently affecting the grassland carrying capacity for livestock^81–83^. Elevated precipitation levels increase above-ground biomass, enabling herders to maintain more livestock at these productive sites, where forage consumption is higher^84^. Furthermore, few studies have shown high grassland carrying capacities during summer, owing to the high foraging value in relatively warm and humid conditions^81,85^. The strong relationship between livestock, human population density and proximity to settlements reflects (i) the huge demand for meat in the rural-urban continuum, (ii) the need for people and infrastructure to raise livestock, and (iii) the importance of KZ as an exportation hub^2,32,75,86^. Larger livestock herds in KZ are generally mobile, traveling to remote locations during the summers and sometimes moving to remote bases in the winter months. Small to medium livestock herds are more likely to be housed in and around the settlements and locations with water access for feeding or grazing^32,52,87,88^. For centuries, pump wells were an integral component of grazing migrations in KZ. Thousands of new pump wells and outposts that serve as seasonal base camps were constructed during the Soviet era to support the regulated migrations of larger livestock herds. However, after the Soviet Union collapsed, most of these outposts were abandoned, and the wells deteriorated^15,89,90^. We could not consider wells and outposts in modeling LSK_D_ distributions because of a lack of national data on the location of working wells or occupied outposts. Hence, we considered the distance to water bodies as a proxy of surface water availability, but it did not result as an important driver in our modeling. This might be because the water body locations used in the current study are derived from the Landsat satellite series that detects and counts a small patch of water area as a water body^43^. This generated ~0.2 million water body locations across KZ (Supplementary Fig. S10), altering proximity distances and true water sources for livestock.

### Limitations and assumptions

Our modeling framework was developed based on the following assumptions: 1) Generally, larger livestock herds in KZ travel to areas remote from settlements (outside our 10 km buffers) during summers for grazing. However, we have limited our buffer distances to 10 km around the settlements that account for the daily mobility of livestock from villages returning at night. 2) Although livestock in KZ follows transhumance concerning seasons, the models were developed with annual livestock census data and did not consider seasonal movements of livestock. 3) We selected ten settlement locations per district, assuming that livestock co-exists with the human population and are housed in and around the settlements. However, some livestock are housed at remote bases well outside settlements. 4) We selected ten settlements per district from the available 100 or 1000 settlement points with a high human population. However, larger herds may be available in rural areas with minimal human population. 5) We assigned the same livestock density values to all 10 settlement locations in a district. However, in reality, livestock densities depend on farm type, availability of forage, water, and other biophysical characteristics in and around the settlements.

The current study has the following limitations: 1) KZ has a national livestock database linked to the national identification program, where every owner must declare each animal owned, all births, deaths, and sales. KZ also has sub-district or village-level livestock counts tagged with geographical information. However, the database is not publicly available and must be collected from each village/district office, which is tedious and time-consuming, thereby limiting the analysis to district-level input data and preventing model validation at the village level. 2) The lack of seasonal livestock density data at the district or sub-district level limits the analysis of animal movement patterns and seasonal livestock density maps. 3) We used human population settlements as a proxy of livestock farm locations due to the absence of information on the geographical distribution of animal farm units. Despite these limitations, the developed database could provide new insights into public health, sustainable livestock ecosystem-environment management, and food security.

Our work compliments GLW-4 with longitudinal, high-resolution gridded LSK_D_ estimates, a high demonstrated validity, and broad potential utility. Previous studies have used similar livestock-related attributes to (i) determine hotspots of livestock-induced pollution^91^, (ii) enhance accuracy in emission inventory and water/air quality simulations^92–94^, (iii) assess eutrophication potential caused by manure emissions^95,96^, (iv) combine crop-livestock systems to promote circular agriculture^97–99^, etc. We propose that the current approach with limited spatial predictors efficiently explains the spatial and temporal changes of LSK_D_ in KZ. However, other factors related to land cover, demographic changes in farmer population, farm sizes, migration, governance and politics, policy implementation and subsidies, and attractiveness of other sector employment could be considered when modeling LSK_D_ patterns for future years. We did not test the model with more or fewer settlement location points per district, which can be tested in future studies. Different machine learning or deep learning algorithms should be tested in the future to develop livestock density maps. Finally, future studies could also map cattle distributions and differentiate grazing intensity and housing systems by categorizing livestock maintained under agricultural, private, and household farms for multiple years.

## Usage Notes

The database can aid in livestock-related studies, as obtaining sub-national livestock census data is challenging. The current study provides a crucial step forward in understanding LSK_D_ distribution in Kazakhstan. The modeling framework is transformative to other regions when observed livestock census data are available, albeit different split ratios and buffer distances around the ruminant holding locations need to be explored for accurate mapping. The developed livestock database can be used in spatially explicit research, such as quantifying grass-livestock balance, water consumption by animal husbandry, methane emissions, risk of zoonotic diseases and other environmental impact assessments. Both the lessons and database, generated from this study serve as essential foundation for sustainable livestock management, food security and other relevant practices and policy development in Kazakhstan.

### Supplementary information

SUPPLEMENTARY INFORMATION

## Data Availability

The code is fully operational under R 4.0.3. Variable standardization and statistical processing were performed using *dplyr*, *caret*, *mgcv*, *randomForest*, *MLmetrics*, *Kendall*, *trend*, *SpatialEco* and *iml* packages. The full code and sample dataset are publicly available through the *figshare* repository (10.6084/m9.figshare.23528232)^72^.
