# Supplementary material for: Gridded livestock density database and spatial trends for Kazakhstan
Source: Sci Data. 2023 Nov 29;10:839. doi: 10.1038/s41597-023-02736-5 (PMC10687097; doi:10.1038/s41597-023-02736-5)
Supplement: Supplementary file 1 — SUPPLEMENTARY INFORMATION [file 41597_2023_2736_MOESM1_ESM.docx]

**Gridded livestock density database and spatial trends for Kazakhstan**

Venkatesh Kolluru^a, *^, Ranjeet John^a, b^, Sakshi Saraf^b^, Jiquan Chen^c, d^, Brett Hankerson^e^, Sarah Robinson^f^, Maira Kussainova^g,h^, Khushboo Jain^a^

*Corresponding author: Venkatesh Kolluru; Email: [Venkatesh.Kolluru@coyotes.usd.edu](mailto:Venkatesh.Kolluru@coyotes.usd.edu); Orcid: 0000-0002-2110-5560

a. Department of Sustainability and Environment, University of South Dakota, Vermillion, SD 57069, USA

b. Department of Biology, University of South Dakota, Vermillion, SD 57069, USA

c. Department of Geography, Environment, and Spatial Sciences, Michigan State University, East Lansing, MI 48823, USA

d. Center for Global Change and Earth Observations, Michigan State University, East Lansing, MI 48823, USA

e. Leibniz Institute of Agricultural Development in Transition Economies (IAMO), Theodor-Lieser-Str. 2, 06120 Halle (Saale), Germany

f. Institute for Agricultural Policy and Market Research & Centre for International Development and Environmental Research (ZEU), Justus Liebig University, Giessen, Germany

g. Kazakh National Agrarian Research University, AgriTech Hub KazNARU, 8 Abay avenue, Almaty 050010, Kazakhstan

h. Kazakh-German University (DKU), Nazarbaev avenue, 173, 050010 Almaty, Kazakhstan


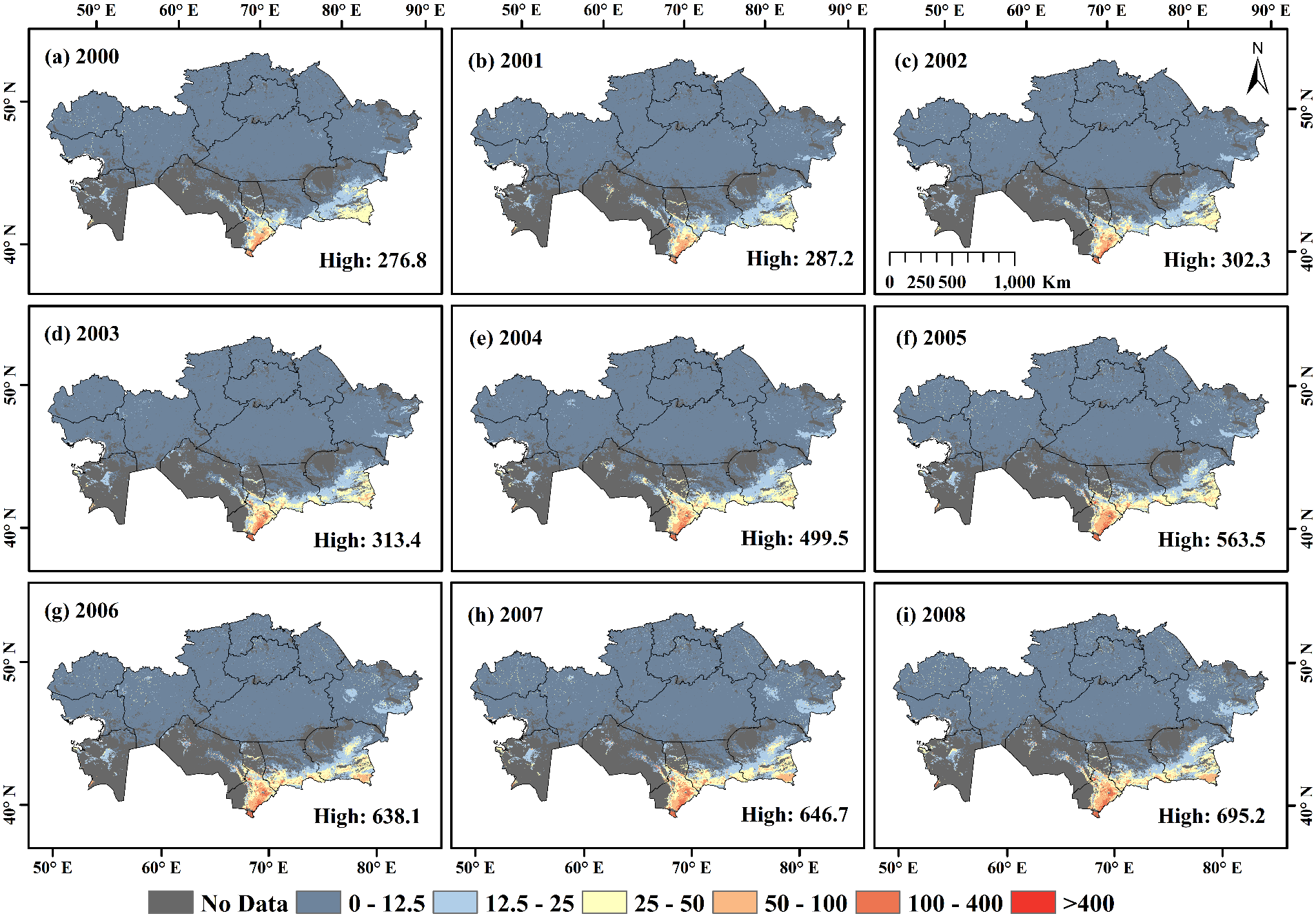


Fig. S1 (a-i) Spatial distribution of estimated small ruminant (sheep & goat) density across Kazakhstan for 2000-2008.


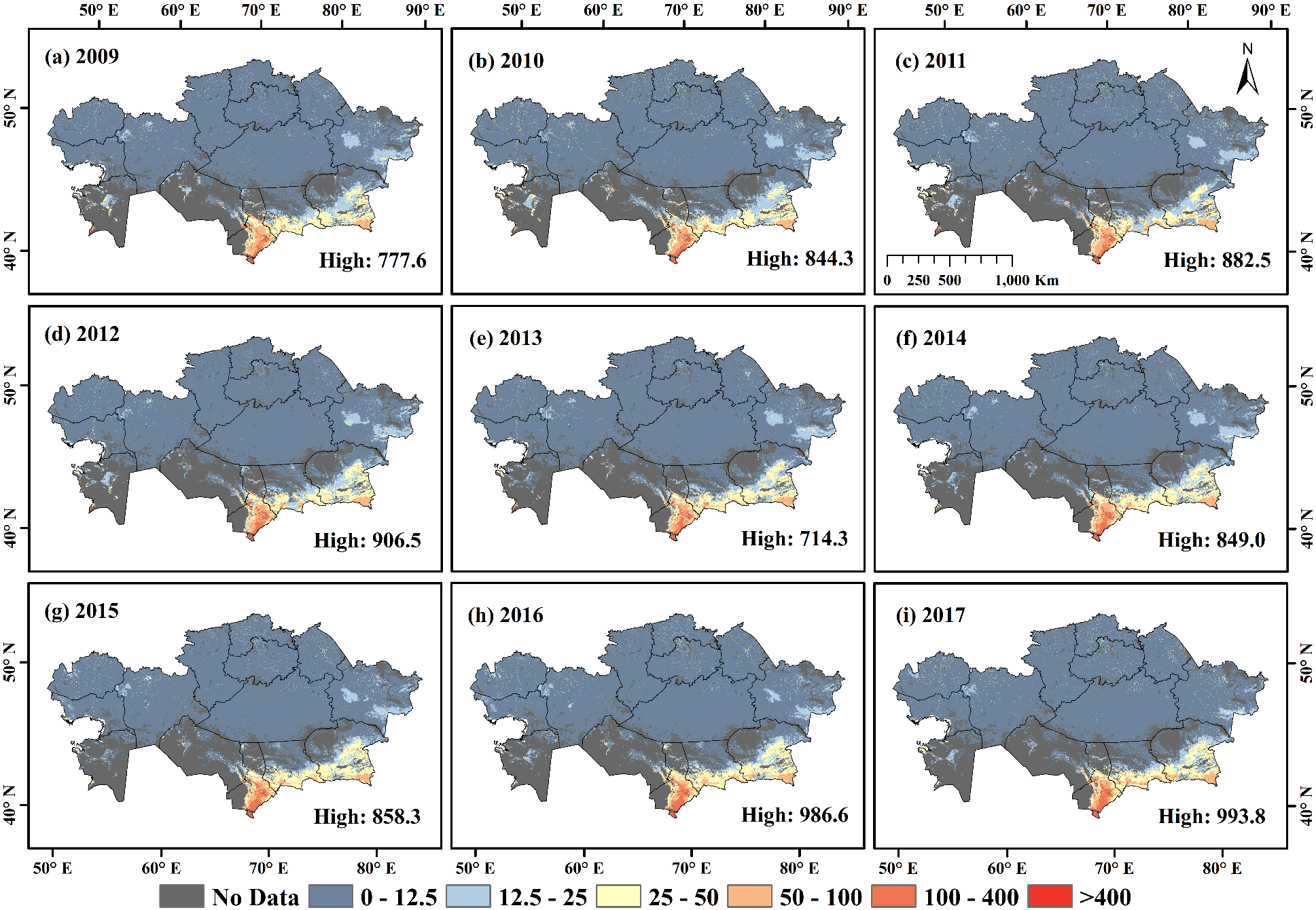


Fig. S2 (a-i) Spatial distribution of estimated small ruminant (sheep & goat) density across Kazakhstan for 2009-2017.


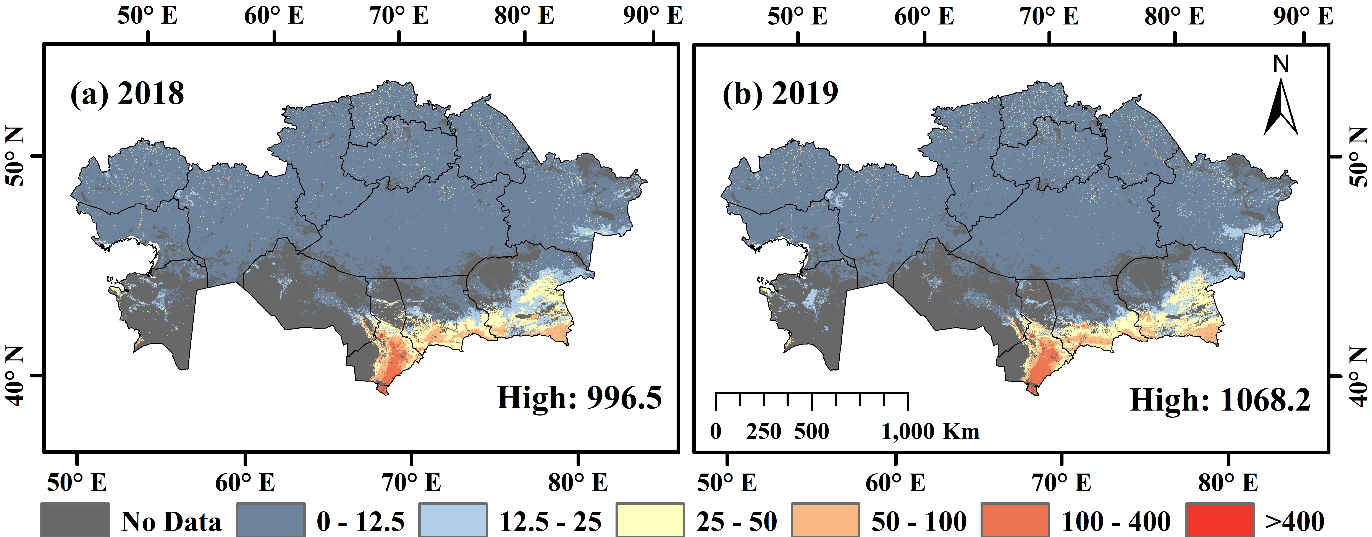


Fig. S3 (a-b) Spatial distribution of estimated small ruminant (sheep & goat) density across Kazakhstan for 2018-2019.


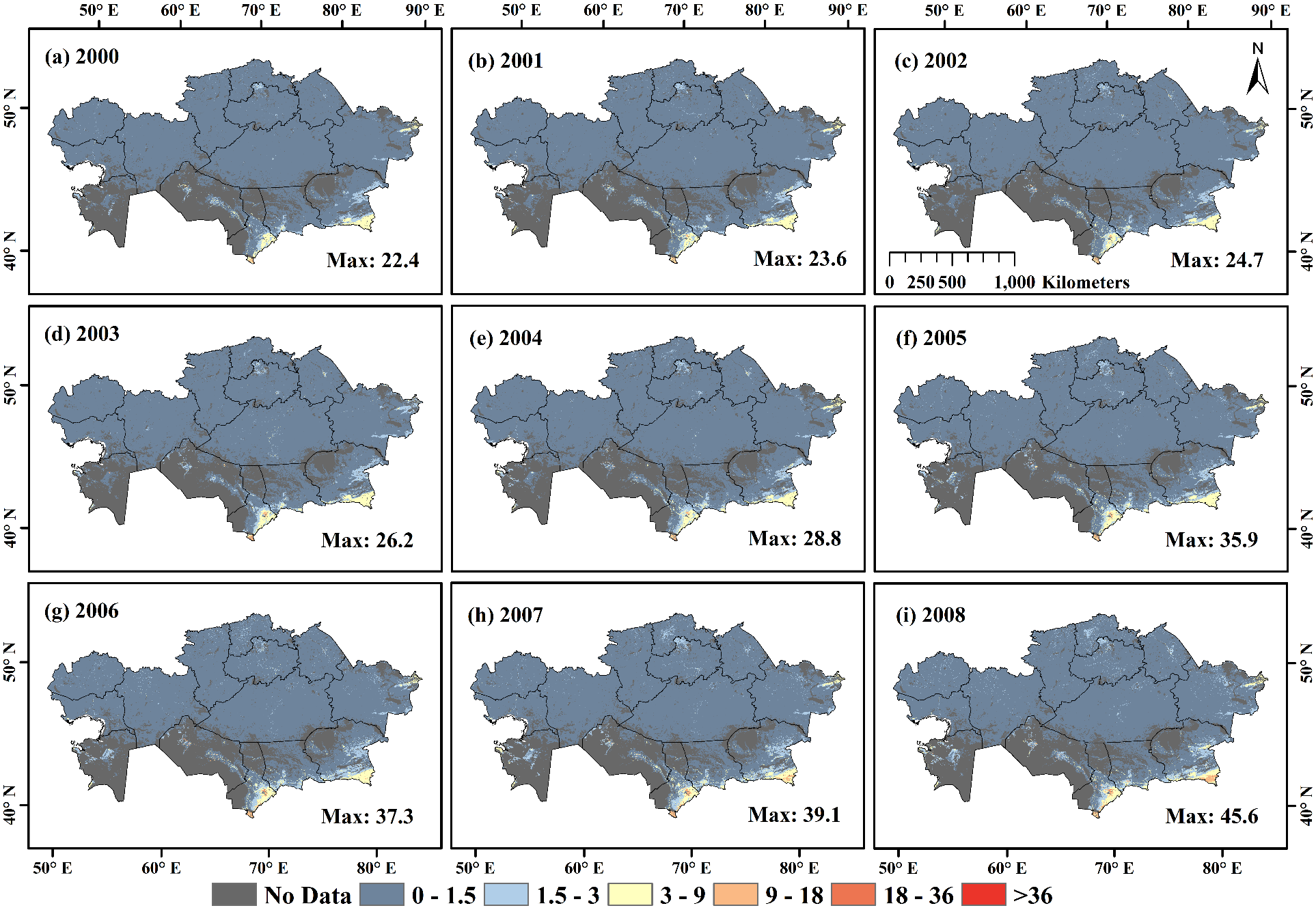


Fig. S4 (a-i) Spatial distribution of estimated horse density across Kazakhstan for 2000-2008.


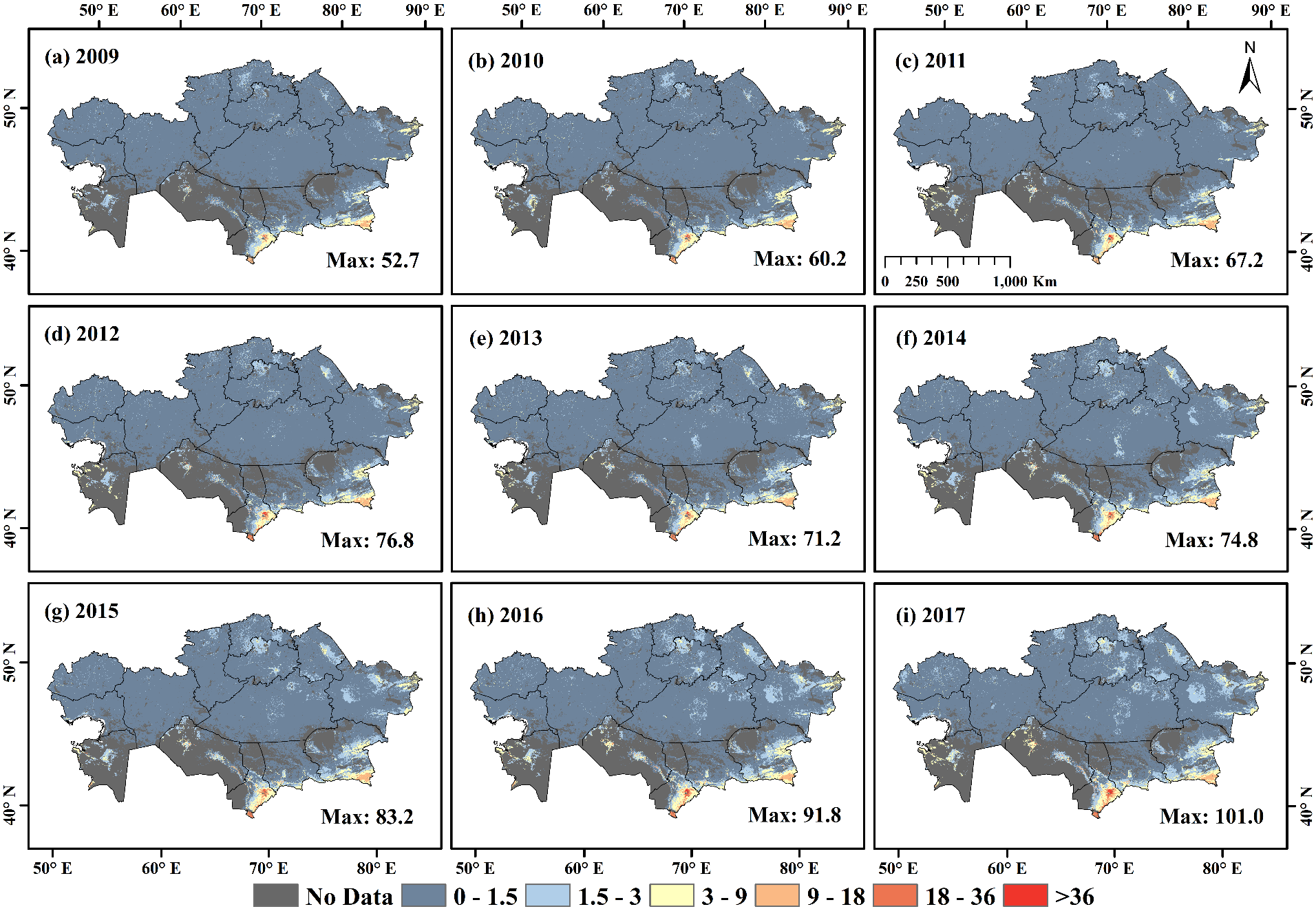


Fig. S5 (a-i) Spatial distribution of estimated horse density across Kazakhstan for 2009-2017.


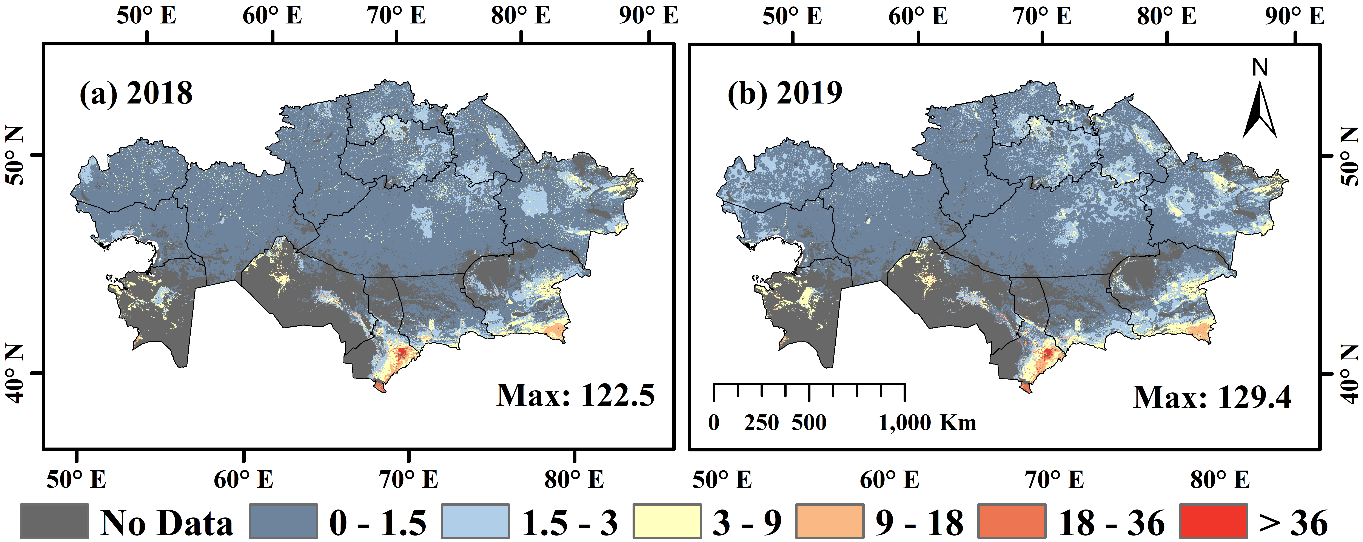


Fig. S6 (a-b) Spatial distribution of estimated horse density across Kazakhstan for 2018-2019.


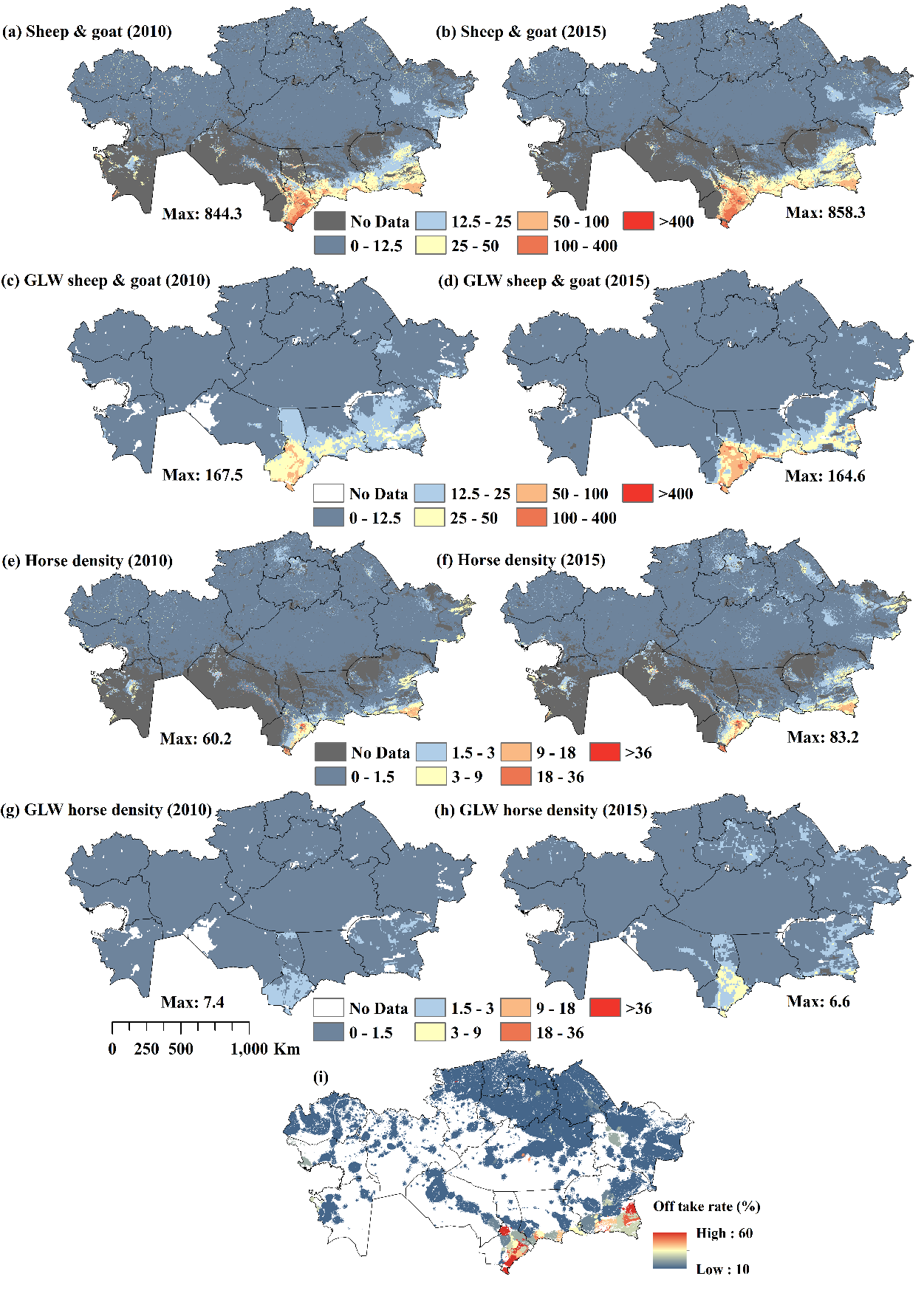


Fig. S7 Small ruminant (sheep & goat) density distributions obtained from this study (a & b) and from Gridded livestock of the world (GLW-3) (c & d) for 2010 & 2015. Horse density distributions obtained from this study (e & f) and GLW-3 (g & h) for 2010 & 2015. (i) Off take rate for 2015 from Hankerson et al. 2019 study. Note: Units are not directly convertible. Though different methods and inputs were used, all maps showed similar distributions of relative grazing activity.


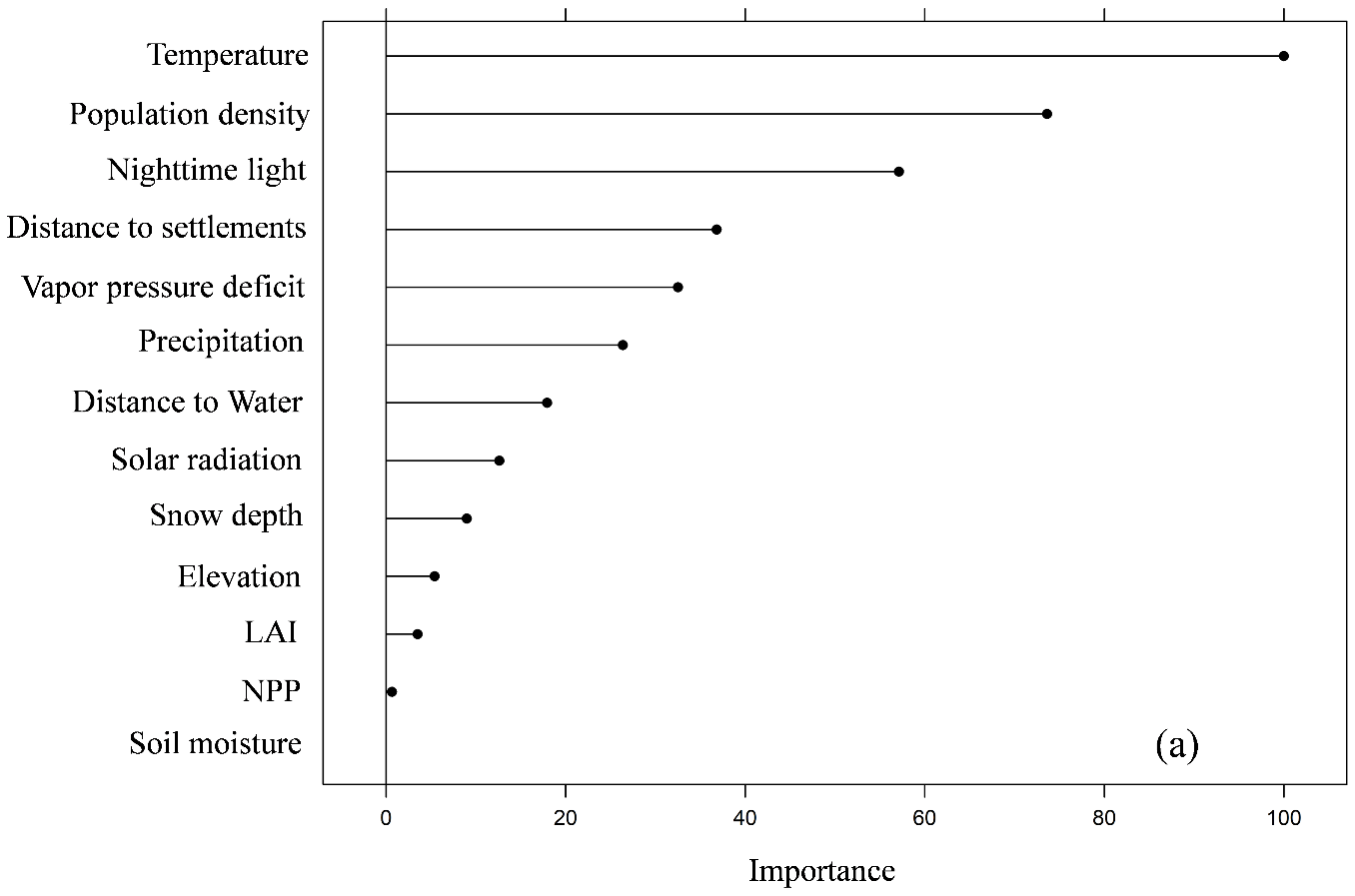


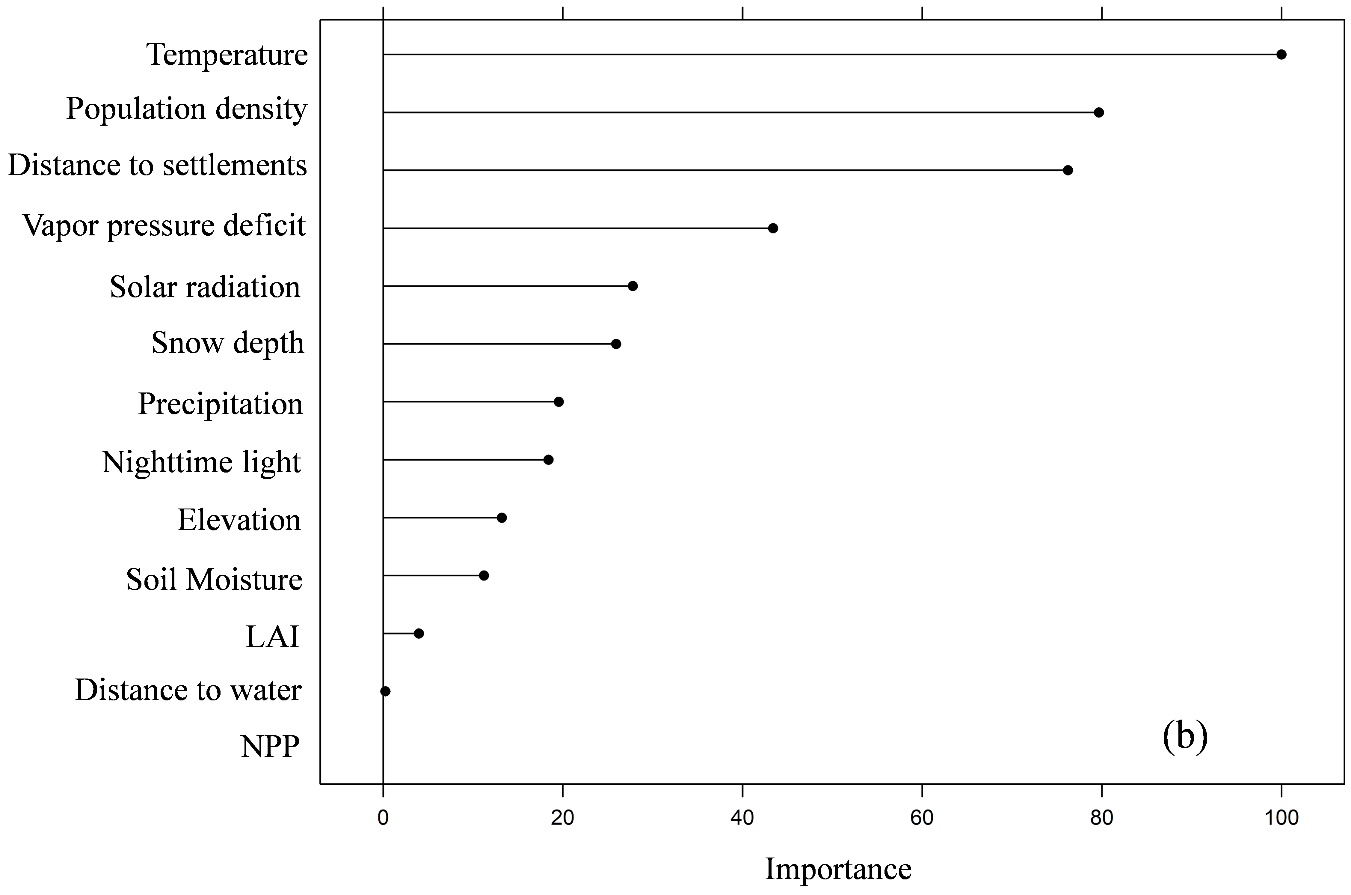


Fig. S8 Variable importance from the random forest regression model for predicting the (a) small ruminant (sheep & goat) and (b) horse density estimates for 2015.


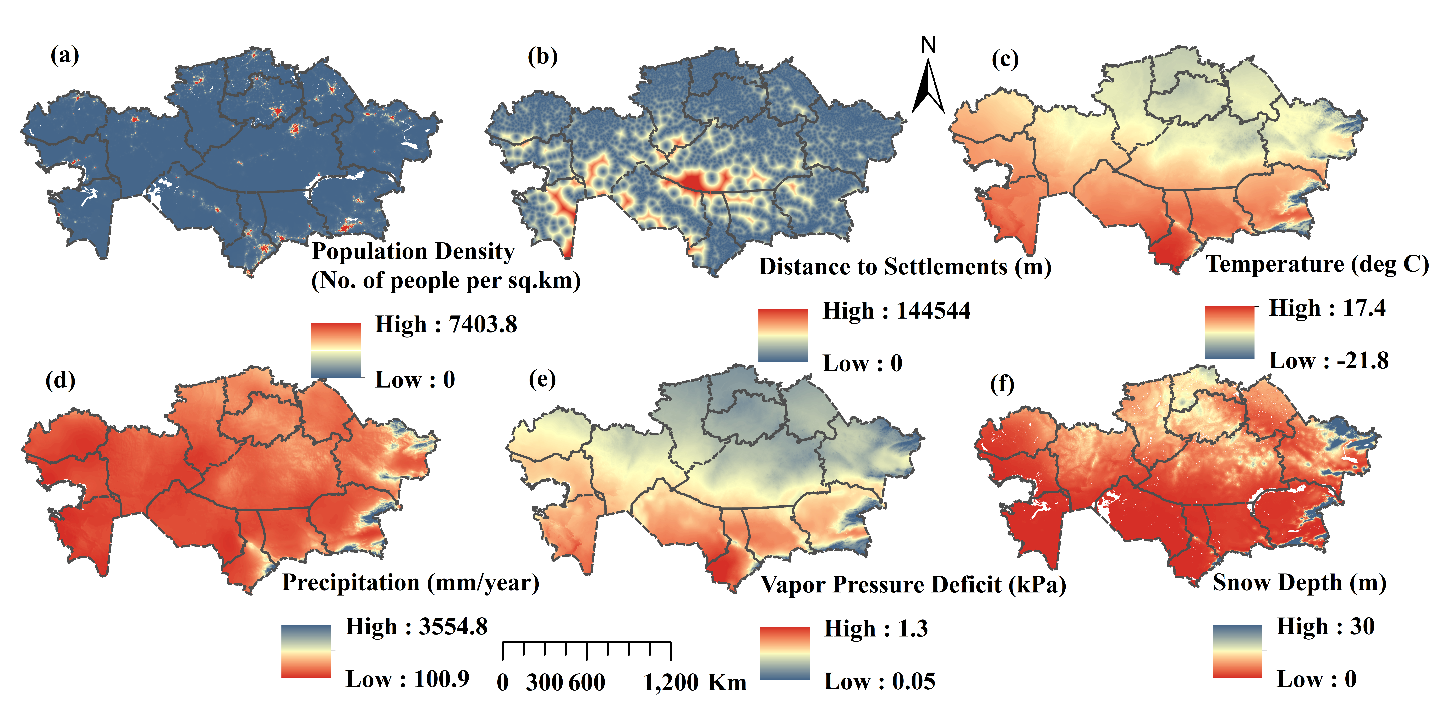


Fig. S9 Socio environmental system drivers used in this study for predicting livestock density estimates in Kazakhstan using random forest regression model.


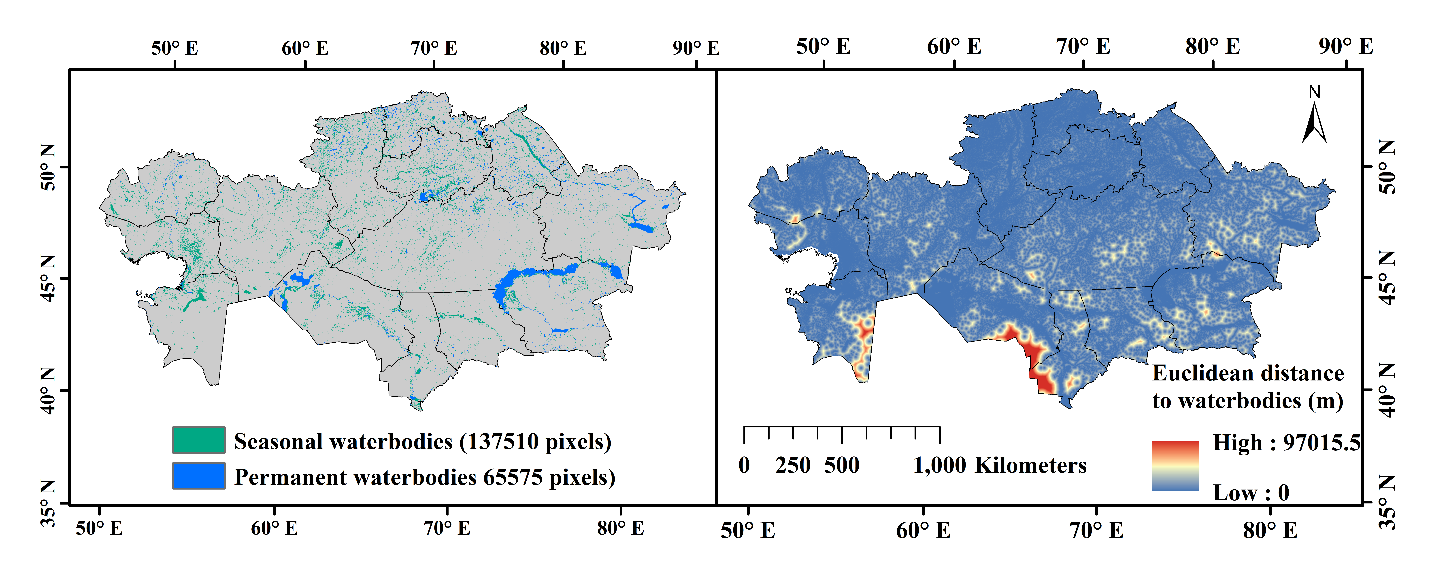


Fig. S10 (a) Seasonal and permanent waterbody locations in 2015 obtained from JRC Yearly Water Classification dataset for Kazakhstan (~0.2 million water body pixels were detected) and (b) Euclidean distance calculated from all the water body locations.

Table S1 Statistical fit metrics of random forest regression model for predicting small ruminant (sheep & goat) and horses density estimates for 2000-2019 (lower rmse/mae and higher R^2^ indicate better model fit). The fit statistics are computed between the observed and predicted livestock density values for the test dataset (10% of the settlement points per year)

| **Year** | **RMSE** | **R^2^** | **MAE** | **RMSE** | **R^2^** | **MAE** |
| --- | --- | --- | --- | --- | --- | --- |
|  | **Small ruminant (sheep and goat) density predictions** | | | **Horse density predictions** | | |
| 2000 | 6.53 | 0.76 | 2.77 | 0.43 | 0.70 | 0.22 |
| 2001 | 5.16 | 0.83 | 2.34 | 0.45 | 0.62 | 0.21 |
| 2002 | 7.37 | 0.79 | 3.11 | 0.62 | 0.58 | 0.26 |
| 2003 | 7.32 | 0.83 | 3.01 | 1.15 | 0.63 | 0.43 |
| 2004 | 7.71 | 0.77 | 3.09 | 0.52 | 0.64 | 0.25 |
| 2005 | 8.54 | 0.85 | 3.70 | 0.60 | 0.76 | 0.26 |
| 2006 | 10.22 | 0.81 | 3.78 | 0.70 | 0.75 | 0.27 |
| 2007 | 9.73 | 0.74 | 4.08 | 0.75 | 0.71 | 0.30 |
| 2008 | 9.01 | 0.87 | 3.82 | 0.63 | 0.75 | 0.27 |
| 2009 | 7.73 | 0.94 | 3.71 | 0.64 | 0.73 | 0.29 |
| 2010 | 10.86 | 0.83 | 4.47 | 0.91 | 0.64 | 0.32 |
| 2011 | 7.78 | 0.93 | 3.85 | 0.88 | 0.64 | 0.34 |
| 2012 | 9.46 | 0.78 | 3.86 | 1.22 | 0.57 | 0.41 |
| 2013 | 10.46 | 0.71 | 4.37 | 1.05 | 0.68 | 0.41 |
| 2014 | 7.36 | 0.88 | 3.49 | 0.78 | 0.80 | 0.37 |
| 2015 | 11.75 | 0.83 | 4.74 | 2.86 | 0.54 | 0.78 |
| 2016 | 8.52 | 0.86 | 3.96 | 1.69 | 0.58 | 0.62 |
| 2017 | 13.34 | 0.63 | 4.17 | 1.96 | 0.57 | 0.60 |
| 2018 | 16.83 | 0.78 | 4.56 | 1.92 | 0.56 | 0.64 |
| 2019 | 18.12 | 0.79 | 4.76 | 1.84 | 0.64 | 0.69 |
